# Superior mesenteric artery syndrome complicated by acute pancreatitis: a case report

**DOI:** 10.1093/jscr/rjag324

**Published:** 2026-04-28

**Authors:** Tetsushi Azami, Yuichi Takano, Suzuno Kazahari, Naoki Tamai, Jun Noda, Fumitaka Niiya, Masatsugu Nagahama

**Affiliations:** Division of Gastroenterology, Department of Internal Medicine, Showa Medical University Fujigaoka Hospital, Yokohama, Kanagawa, Japan; Division of Gastroenterology, Department of Internal Medicine, Showa Medical University Fujigaoka Hospital, Yokohama, Kanagawa, Japan; Division of Gastroenterology, Department of Internal Medicine, Showa Medical University Fujigaoka Hospital, Yokohama, Kanagawa, Japan; Division of Gastroenterology, Department of Internal Medicine, Showa Medical University Fujigaoka Hospital, Yokohama, Kanagawa, Japan; Division of Gastroenterology, Department of Internal Medicine, Showa Medical University Fujigaoka Hospital, Yokohama, Kanagawa, Japan; Division of Gastroenterology, Department of Internal Medicine, Showa Medical University Fujigaoka Hospital, Yokohama, Kanagawa, Japan; Division of Gastroenterology, Department of Internal Medicine, Showa Medical University Fujigaoka Hospital, Yokohama, Kanagawa, Japan

**Keywords:** superior mesenteric artery syndrome, acute pancreatitis, duodenal obstruction

## Abstract

Superior mesenteric artery (SMA) syndrome is a relatively rare condition caused by compression of the third portion of the duodenum between the aorta and the SMA. The coexistence of SMA syndrome and acute pancreatitis is even rarer and remains poorly recognized in clinical practice. A 36-year-old woman with a history of cerebral palsy and scoliosis presented with epigastric pain and abdominal distension. Contrast-enhanced computed tomography revealed compression of the third portion of the duodenum with dilatation of the proximal gastrointestinal tract, leading to the diagnosis of SMA syndrome. In addition, peripancreatic fat stranding was observed, suggesting concomitant acute pancreatitis. Other causes of pancreatitis were considered unlikely. The patient improved rapidly with conservative treatment and was discharged without recurrence. Clinicians should be aware that SMA syndrome may be complicated by acute pancreatitis, particularly in patients with worsening abdominal pain.

## Introduction

Superior mesenteric artery (SMA) syndrome is a relatively rare condition caused by compression of the third portion of the duodenum between the aorta and the SMA. It is typically associated with anatomical or physiological factors such as weight loss and spinal deformities, and commonly presents with symptoms of proximal intestinal obstruction, including abdominal pain, nausea, and vomiting. The condition is more likely to occur in patients with chronic debilitating diseases or poor nutritional status. Although SMA syndrome is a well-recognized cause of duodenal obstruction, its clinical presentation is variable [1].

The coexistence of SMA syndrome and acute pancreatitis is rare, and this association remains poorly recognized in clinical practice.

## Case report

A 36-year-old woman with a history of cerebral palsy and scoliosis presented to our hospital with a 2-day history of epigastric pain and abdominal distension. Her height was 142 cm and body weight was 40 kg (body mass index: 19.8 kg/m^2^). On physical examination, abdominal distension and epigastric tenderness were noted without rebound tenderness. Vital signs were stable, and there were no signs of fever, shock, or organ failure.

**Figure 1:**
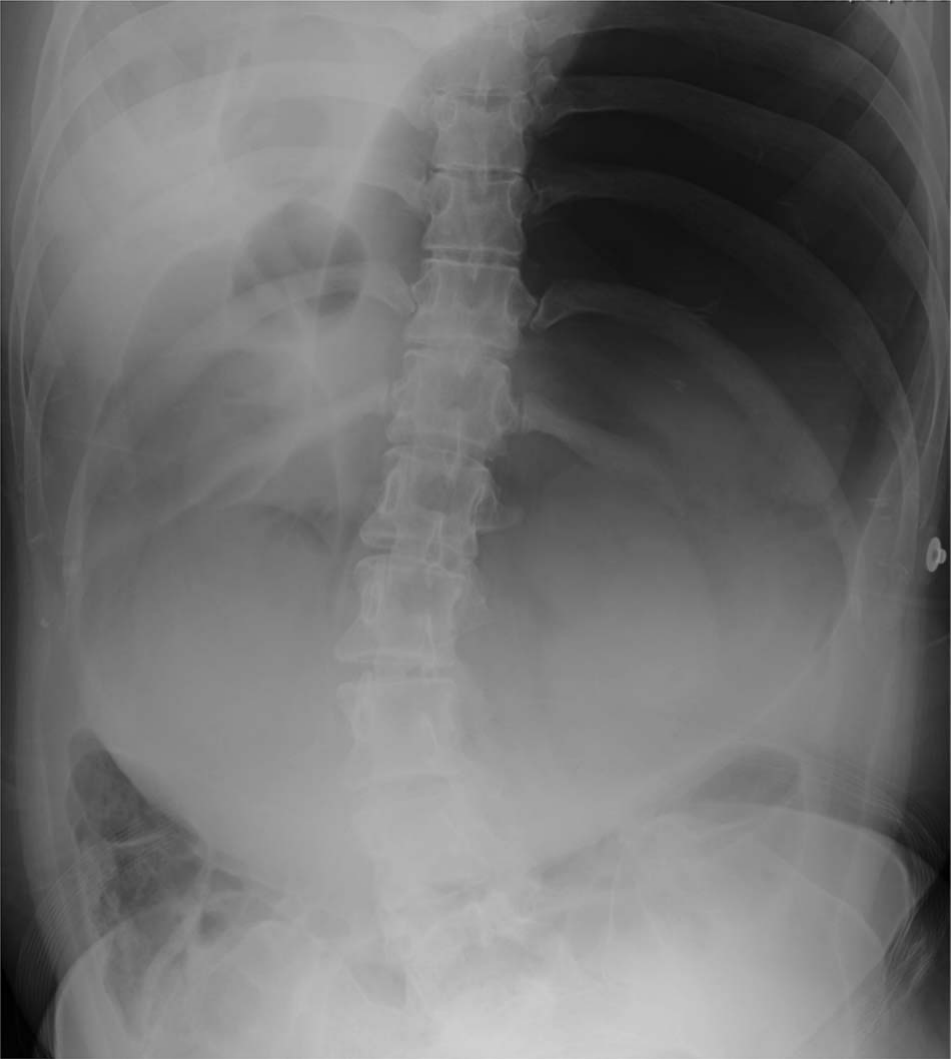
Abdominal radiograph showing marked gastric dilatation, suggesting proximal intestinal obstruction.

**Figure 2:**
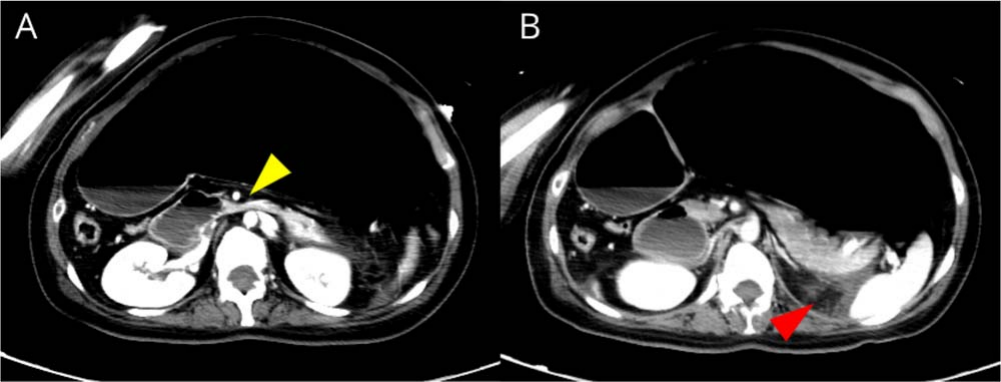
(A) Axial contrast-enhanced computed tomography image showing compression of the third portion of the duodenum between the aorta and the superior mesenteric artery, with marked proximal dilatation of the stomach and duodenum. (B) Contrast-enhanced computed tomography image showing peripancreatic fat stranding extending to the inferior pole of the left kidney, without evidence of pancreatic necrosis.

Laboratory tests revealed elevated pancreatic amylase (783 U/L) and C-reactive protein (22.8 mg/dl). A mild increase in white blood cell count was observed, while hepatobiliary enzymes were within normal limits. Abdominal radiography demonstrated marked gastric dilatation (Fig. 1). Contrast-enhanced computed tomography showed compression of the third portion of the duodenum between the aorta and the SMA, with marked dilatation of the stomach and proximal duodenum. The aortomesenteric distance was reduced to 7 mm, consistent with SMA syndrome [1, 2]. Contrast-enhanced computed tomography demonstrated inflammatory fat stranding extending from the pancreatic body and tail to the inferior pole of the left kidney, without evidence of pancreatic necrosis (Fig. 2). These findings were consistent with interstitial edematous pancreatitis. Based on the overall clinical course and imaging findings, the severity of acute pancreatitis was classified as moderately severe according to the revised Atlanta classification [3]. No gallstones or biliary dilatation were identified. There was no history of alcohol use, and other causes of pancreatitis, including drug-induced and hypertriglyceridemia-related pancreatitis, were considered unlikely.

**Figure 3:**
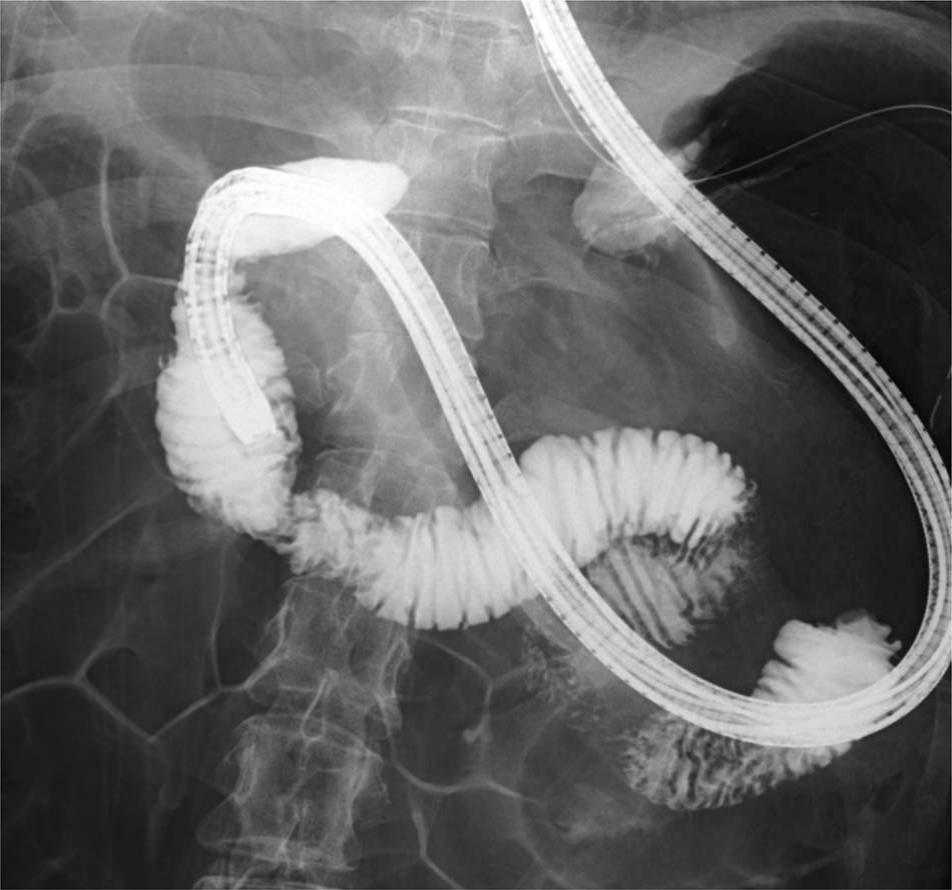
Endoscopic duodenography demonstrating no evidence of fixed stenosis or obstructive lesion in the third portion of the duodenum.

Based on these findings, the patient was diagnosed with SMA syndrome complicated by acute pancreatitis. She was treated conservatively with nasogastric decompression and intravenous fluid therapy. Her symptoms improved rapidly, and inflammatory markers gradually decreased. Endoscopic duodenography showed no evidence of fixed stenosis or neoplastic lesions in the third portion of the duodenum (Fig. 3). Oral intake was resumed without recurrence of symptoms, and the patient was discharged.

## Discussion

Imaging findings are essential for the diagnosis of SMA syndrome. In particular, a reduced aortomesenteric distance and dilatation of the proximal gastrointestinal tract are considered characteristic features. In the present case, the diagnosis was established based on these typical imaging findings in combination with clinical symptoms and medical history.

Several mechanisms have been proposed to explain the development of acute pancreatitis in SMA syndrome. The most widely accepted hypothesis is that duodenal obstruction leads to increased intraduodenal pressure, resulting in impaired pancreatic duct outflow. This may cause pancreatic juice stasis and subsequent activation of pancreatic enzymes. Additionally, retrograde reflux into the pancreatic duct due to elevated intraduodenal pressure has also been suggested. These mechanisms may act in combination to induce pancreatitis. Although these pathophysiological explanations are plausible, the condition remains rare, and only a limited number of cases with detailed clinical information have been reported [3–8].

A review of five previously reported cases together with the present case (total of six cases) showed a median age of 27 years (range, 18–76 years), with four females and two males. The median BMI was 15.4 kg/m^2^ (range, 14.5–19.8 kg/m^2^), and most patients had a low BMI. The severity of pancreatitis varied, including one mild, two moderate, and three severe cases. All patients were treated conservatively, and all showed clinical improvement.

In the present case, the BMI was relatively preserved compared with previously reported cases; however, scoliosis may have contributed as an anatomical predisposing factor for SMA syndrome. Therefore, this condition should not be considered limited to patients with low BMI.

In clinical practice, attention is often focused on symptoms of intestinal obstruction in SMA syndrome, and the coexistence of acute pancreatitis may be overlooked. In patients presenting with worsening abdominal pain or elevated inflammatory markers, the possibility of this complication should be considered.

## Conclusion

Although rare, SMA syndrome may be complicated by acute pancreatitis, and awareness of this association is important. In particular, clinicians should consider this complication in patients with worsening abdominal pain or increasing inflammatory markers.
